# Shrinkage regression-based methods for microarray missing value imputation

**DOI:** 10.1186/1752-0509-7-S6-S11

**Published:** 2013-12-13

**Authors:** Hsiuying Wang, Chia-Chun Chiu, Yi-Ching Wu, Wei-Sheng Wu

**Affiliations:** 1 Institute of Statistics, National Chiao Tung University, 1001 University Road, 300 Hsinchu, Taiwan (R. O. C; 2 Department of Electrical Engineering, National Cheng Kung University, No.1 University Road, 701 Tainan, Taiwan

**Keywords:** missing value, imputation, microarray, regression

## Abstract

**Background:**

Missing values commonly occur in the microarray data, which usually contain more than 5% missing values with up to 90% of genes affected. Inaccurate missing value estimation results in reducing the power of downstream microarray data analyses. Many types of methods have been developed to estimate missing values. Among them, the regression-based methods are very popular and have been shown to perform better than the other types of methods in many testing microarray datasets.

**Results:**

To further improve the performances of the regression-based methods, we propose shrinkage regression-based methods. Our methods take the advantage of the correlation structure in the microarray data and select similar genes for the target gene by Pearson correlation coefficients. Besides, our methods incorporate the least squares principle, utilize a shrinkage estimation approach to adjust the coefficients of the regression model, and then use the new coefficients to estimate missing values. Simulation results show that the proposed methods provide more accurate missing value estimation in six testing microarray datasets than the existing regression-based methods do.

**Conclusions:**

Imputation of missing values is a very important aspect of microarray data analyses because most of the downstream analyses require a complete dataset. Therefore, exploring accurate and efficient methods for estimating missing values has become an essential issue. Since our proposed shrinkage regression-based methods can provide accurate missing value estimation, they are competitive alternatives to the existing regression-based methods.

## Background

Nowadays microarray technique has become an important and useful tool in functional genomics research. This high throughput technique allows the characterization of the gene expression of the whole genome by measuring the relative transcript levels of thousands of genes in various experimental conditions or time points [1]. Microarray data analyses have been widely used to investigate various biological processes such as the cell cycle process [2-8] and the stress response [9,10].

Although the microarray technology has been developed for more than a decade, typical microarray data still contain more than 5% missing values with up to 90% of genes affected [11]. Missing values could be generated by various reasons, including technological failures, administrative error, insufficient resolution, image corruption, dust or scratches on the slide [12]. As many downstream analysis methods (such as gene clustering, disease classification and gene network reconstruction) require complete datasets, missing value estimation becomes an important pre-processing step in the microarray data analysis [11-13].

The missing values in the microarray dataset are traditionally estimated by repeating the microarray experiments or simply replacing the missing values with zero or the row average (the average expression over the experimental conditions). Because these approaches are either time-consuming or leading to serious estimation errors, more advanced missing value imputation methods are needed to solve the missing value problems. In 2001, Troyanskaya *et al. *published the first two missing value imputation algorithms based on the k-nearest neighbors (kNNimpute) and the singular value decomposition (SVDimpute) [12]. Since then, a lot of missing value imputation methods have been proposed such as *Bayesian *principal component analysis (BPCA) [14], Gaussian mixture clustering imputation (GMCimpute) [11], conditional ordered list imputation [15], random-forest-based imputation [16] and so on.

Among the existing missing value imputation methods, the regression-based methods are very popular and contain many algorithms, including least squares imputation (LSimpute) [17], local least squares imputation (LLSimpute) [18], sequential local least squares imputation (SLLSimpute) [19], and iterated local least squares imputation (ILLSimpute) [13]. LSimpute estimates the missing values in the target gene by using a weighted average of the *k *estimates from the *k *most similar genes. Each estimate is attained by constructing a single regression model of the target gene by a similar gene. LLSimpute represents the target gene as a linear combination of *k *similar genes by a multiple regression model and uses the regression coefficients to estimate the missing values. SLLSimpute modifies the LLSimpute by estimating the missing values sequentially from the gene containing the fewest missing values and partially utilizing these estimated values. ILLSimpute modifies the LLSimpute by not choosing the similar genes with a fixed number *k *but defining the similar genes as the genes whose distances from the target gene are less than a distance threshold and then runs LLSimpute iteratively.

In this study, we focus on the regression-based methods because these methods have been shown to have better performances than the other existing methods in many testing microarray datasets [20,21]. To further improve the performance of the regression-based methods, we propose shrinkage regression-based methods which use a shrinkage estimator to replace the least square estimator for the estimation of the regression coefficients in the regression model. The shrinkage estimator such as the James-Stein estimator has been shown to dominate the least square estimator in many statistical models [22,23]. By adopting our new regression coefficients in the regression-based methods, we showed that an improvement on missing value estimation in six testing microarray datasets could be achieved.

## Methods

**Figure 1:**
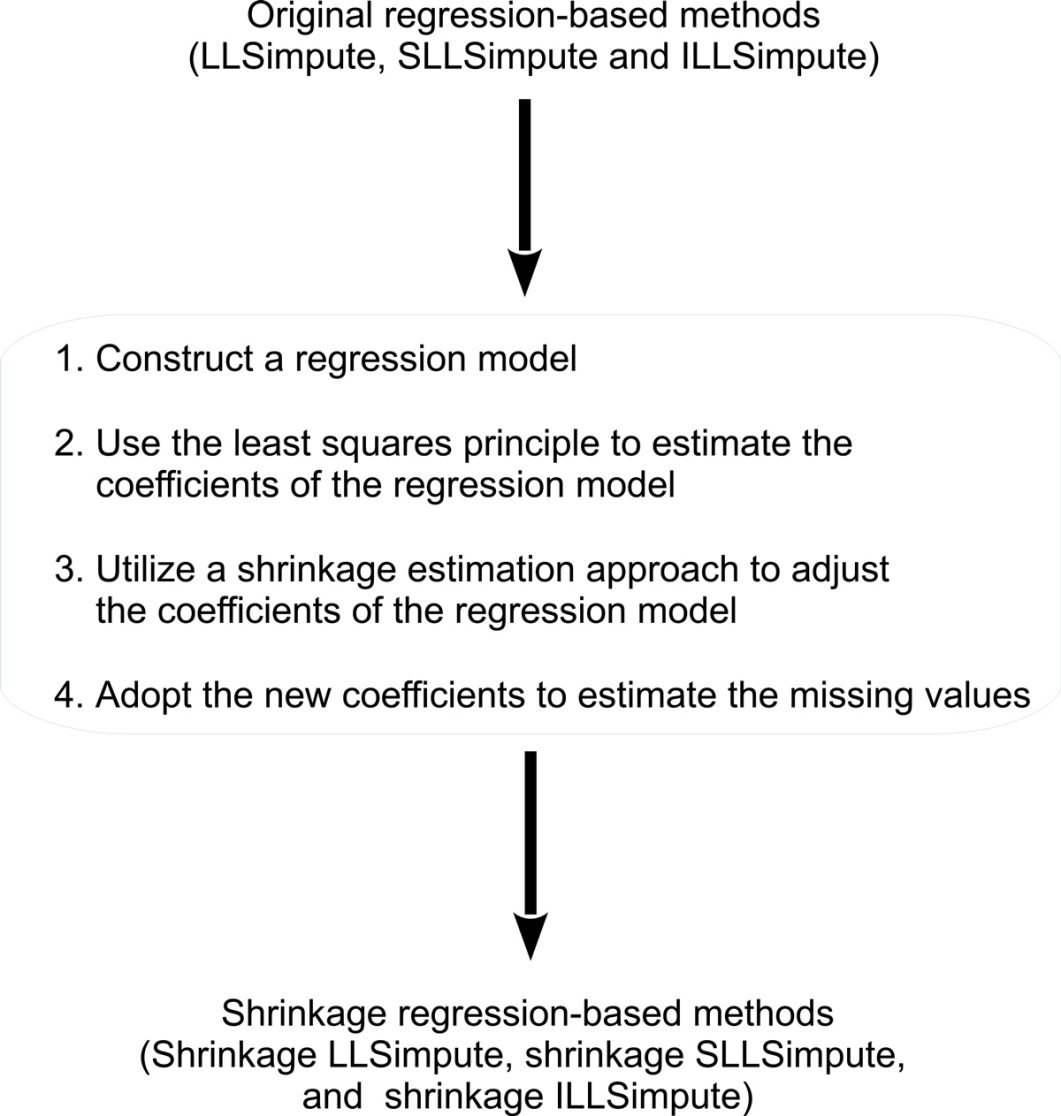
**The shrinkage regression-based methods**.

In this study, we propose using the well-known shrinkage estimation approach to improve three existing regression-based methods (LLSimpute [18], SLLSimpute [19], and ILLSimpute [13]) for missing value estimation. We call our proposed methods the shrinkage regression-based methods (see Figure 1). In the following subsections, we first introduce the shrinkage estimation approach and then describe the proposed shrinkage LLSimpute, shrinkage SLLSimpute, and shrinkage ILLSimpute.

### Shrinkage estimation approach

One of the shrinkage estimators, the James-Stein estimator, for the normal distribution is introduced here. Suppose that *Y*_1_, *Y*_2_, ..., *Y_k _*are independent normal random variables and these *k *random variables all have a common known variance, but their means are unknown and different. Let *Y_i _*~ *N*(*θ_i_*, *σ*^2^) and **Y **= (*Y*_1_, ..., *Y_k_*). Then we have **Y **~ *N*(***θ***, *σ*^2^**I**), where ***θ ***= (*θ*_1_, ..., *θ_k_*) and **I **is a *k *× *k *identity matrix. Let **d**(**Y**) = (*d*_1_(**Y**), ..., *d_k_*(**Y**)) be an estimator of ***θ*. **Under the squared error loss function

(1) $$L\left(\theta -d\left(Y\right)\right)=\overset{k}{\underset{i=1}{\sum }}{\left(\theta_{i}-d_{i}\left(Y\right)\right)}^{2}={||\theta -d\left(Y\right)||}^{2},$$

we are interested in finding estimators of ***θ ***such that the mean squared error *E*_**Y **_[*L *(***θ***, **d**(**Y**))] is minimized. An intuitive estimator of ***θ ***is **Y **(i.e. ${\hat{\theta }}_{i}=Y_{i},\;i=1,\;\ldots ,\;k$). However, Stein [22] showed that when *k *≥ 3, there exists other estimators with smaller mean squared error than the intuitive estimator **Y**. For *k *≥ 3, under the squared error loss, the intuitive estimator **Y **is dominated by the estimator

(2) $${\hat{\theta }}^{JS}=\left(1-\frac{k-2}{S_{Y}^{2}}\right)Y,$$

where $S_{Y}^{2}=\sum_{i=1}^{k}Y_{i}^{2}$[23]. The estimator in (2) is called the James-Stein estimator in the literature [23]. With the form in (2), the James-Stein estimator of *θ_i _*is

(3) $${\hat{\theta }}_{i}^{JS}=\left(1-\frac{k-2}{S_{Y}^{2}}\right)Y_{i}.$$

It is worth noting that the estimator of *θ_i _*in (3) depends on not only the random variable *Y_i_*, but also the other variables *Y*_1_, ..., *Y_i_*_-1_, *Y*_*i *+ 1_, ..., *Y_k _*because of the term $S_{Y}^{2}$. On the contrary, the intuitive estimator ${\hat{\theta }}_{i}=Y_{i}$ does not use the other variables *Y*_1_, ..., *Y*_*i *- 1_, *Y*_*i *+ 1_, ..., *Y_k _*but only uses *Y_i _*to estimate *θ_i_*. It has been shown that estimators using other variables' information provide more accurate estimation for ***θ ***than the intuitive estimator does [22]. In fact, except for the estimator in (3), the estimators of the form

(4) $${\hat{\theta }}_{i}^{JS}=\left(1-\frac{c}{S_{Y}^{2}}\right)Y_{i}$$

all have uniformly smaller mean squared error than the intuitive estimator *Y_i_*, for *k *≥ 3 and 0 <*c <*2 (*k - *2). Among all the estimators of the form in (4), the estimator in (3) has the minimized mean squared error. The shrinkage estimation approach has also been shown to have good performance in interval estimation [24,25]. Based on the James-Stein estimator in (3), we developed shrinkage regression-based imputation methods.

### Notations

In a typical microarray data matrix, the rows are the genes under investigation and the columns are the experimental conditions or time points. The microarray data matrix is obtained by performing a series of experiments on the same set of genes. We use $G\in {\mathbb{R}}^{m\;\times \;n}$ to represent a microarray data matrix with *m *genes and *n *experiments, and assume m≫n which is true for microarray data. In the matrix **G**, a row ${\text{g}}_{i}^{T}\in {\mathbb{R}}^{1\;\times \;n}$ represents the expressions of the *i*th gene in *n *experiments:

(5) $$\text{G}=\left(\begin{array}{c} {\text{g}}_{1}^{T}\\ \vdots \\ {\text{g}}_{m}^{T} \end{array}\right)\in {\mathbb{R}}^{m\;\times n}$$

where ${\text{g}}_{i}^{T}$ denotes the transpose of a column vector **g_i_**. If there is a missing value in the *l*th position of the *i*th gene, we denote it as , i.e. *G_i_*,_*l *_= *g_il _*= .

### Shrinkage local least squares imputation (Shrinkage LLSimpute)

In the LLSimpute method [18], a target gene with missing values is represented as a linear combination of *k *similar genes. Rather than using all genes in the dataset, only *k *genes with high similarity to the target gene are used. The procedure of selecting *k *similar genes is as follows. Suppose that the target gene is the first gene and has a missing value α  in the first position, i.e. α = *g*_11 _in the matrix $G\in {\mathbb{R}}^{m\;\times \;n}$. The Pearson correlation coefficient is used to find the *k *similar genes. These *k *similar genes are called the *k*-nearest neighbor genes, which have the *k *largest absolute values of the Pearson correlation coefficients. The Pearson correlation coefficient $r_{{}_{1j}}$ between the target gene and the *j*th gene is defined as

(6) $$r_{1j}=\frac{1}{n-2}\overset{n}{\underset{t=2}{\sum }}\left(\frac{g_{1t}-{ḡ}_{1}}{\sigma_{1}}\right)\left(\frac{g_{jt}-{ḡ}_{j}}{\sigma_{j}}\right)$$

where ${ḡ}_{j}$ and *σ_j _*denote the average and the sample standard deviation of the vector (*g*_*j*2_, ..., *g_jn_*). When computing the correlation coefficients, *g*_*j*1 _is not used because it corresponds to the position of the missing value in the target gene. Based on these selected *k*-nearest neighbor genes, a matrix $A\in {\mathbb{R}}^{k\;\times \;\left(n-1\right)}$ and two vectors $b\in {\mathbb{R}}^{k\;\times \;1}$ and $\text{w}\in {\mathbb{R}}^{\left(n-1\right)\;\;\times \;\;1}$ can be formed as follows

$$\left(\begin{array}{c} {\text{g}}_{1}^{T}\\ {\text{g}}_{S_{1}}^{T}\\ \vdots \\ {\text{g}}_{S_{k}}^{T} \end{array}\right)=\left(\begin{array}{cc} \alpha & {\text{w}}^{T}\\ \text{b} & \text{A} \end{array}\right)\;=\left(\begin{array}{ccccc} \alpha & w_{1} & w_{2} & & w_{n-1}\\ b_{1} & A_{1,1} & A_{1,2} & & A_{1,n-1}\\ \vdots & \vdots & \vdots & \ldots & \vdots \\ b_{k} & A_{k,1} & A_{k,2} & & A_{k,n-1} \end{array}\right)$$

where α is the missing value in the target gene ***g*_1 _**and $g_{s_{1}},\ldots ,g_{s_{k}}$ are the k-nearest nieghbor genes of the target gene g_1_. Each row of matrix **A **consists of the last *n - *1 elements of one *k*-nearest neighbor gene $g_{s_{i}}$, 1 ≤ *i *≤ *k*. The elements of the vector **b **comprise of the first elements of all these *k*-nearest neighbor genes and the elements of the vector **w **are the last *n - *1 elements of the target gene **g**_1_. With the matrix **A**, and the vectors **b **and **w**, the least squares problem is formulated in LLSimpute as

(7) $$\underset{\text{x}}{\text{min}}{||A^{T}\text{x}-w||}_{2}.$$

Solving the above problem, the least square regression coefficients $\hat{x}\in {\mathbb{R}}^{k\times 1}$ are acquired as

(8) $$\hat{\text{x}}≜{\left({\hat{x}}_{1},{\hat{x}}_{2},\;\ldots ,\;{\hat{x}}_{k}\right)}^{T}={\left(AA^{T}\right)}^{-1}A\text{w}.$$

In the LLSimpute, the missing value is then estimated by

(9) $$\alpha =b^{T}\hat{\text{x}}={\hat{x}}_{1}b_{1}+{\hat{x}}_{2}b_{2}+\ldots +{\hat{x}}_{k}b_{k}.$$

In this study, we want to improve the performance of LLSimpute by adjusting the regression coefficients in (8). Our shrinkage LLSimpute associates the LLSimpute method with the shrinkage estimator to impute the missing values. Our method replaces the regression coefficient estimators $\hat{\text{x}}$ in (8) by the shrinkage estimator, and then use the new estimator to estimate the missing value α in (9). However, we found that applying the existing shrinkage estimator in (3) did not always improve the performance of LLSimpute. Therefore, we tested different forms of the shrinkage coefficient estimators and conceived a feasible coefficient estimator to improve the LLSimpute method. We proposed using the shrinkage regression coefficients

(10) $${\hat{x}}_{i}^{JS}=\left(1-\frac{\left(k-2\right)\sigma^{2}}{ñS^{2}}\right){\hat{x}}_{i}$$

to replace the conventional coefficients in (8), where *σ*^2 ^is the variance of the coefficients $\left({\hat{x}}_{1},{\hat{x}}_{2},\;\ldots ,\;{\hat{x}}_{k}\right),$ S is the norm of the coefficients (i.e. $S^{2}=\sum_{i=1}^{k}{\hat{x}}_{i}^{2}$), *k *is the row number of the matrix **A **and ñ  is the column number of the matrix **A**, which equals *n *- 1 in this case. Finally, the missing value is estimated as

(11) $$\alpha =b^{T}{\hat{\text{x}}}^{JS}={\hat{x}}_{1}^{JS}b_{1}+{\hat{x}}_{2}^{JS}b_{2}+\ldots +{\hat{x}}_{k}^{JS}b_{k}$$

where ${\hat{\text{x}}}^{JS}={\left({\hat{x}}_{1}^{JS},\;\ldots ,\;{\hat{x}}_{k}^{JS}\right)}^{T}$.

### Shrinkage sequential local least squares imputation (Shrinkage SLLSimpute)

In the LLSimpute, it does not use the information of genes with missing values since the existence of missing values hinders the use of the other observed values of that gene. In the SLLSimpute method, it estimates the missing values sequentially from the gene containing the fewest missing values and partially utilizes these estimated values. The details of SLLSimpute [19] is described as follow. First, the microarray matrix $G\in {\mathbb{R}}^{m\;\times \;n}$ is divided into two submatrices: a complete matrix $G_{1}\in {\mathbb{R}}^{m_{1}\times n}$ consisting of genes without missing values and an incomplete matrix $G_{2}\in {\mathbb{R}}^{\left(m-m_{1}\right)\;\times n}$ consisting of genes with missing values. In the incomplete matrix **G**_2_, the genes are sorted by their missing rates. The first gene has the smallest missing rate and the last gene has the largest missing rate. The missing rate is calculated by

(12) $$r_{i}=\frac{c_{i}}{n},$$

where *c_i _*is the number of missing values in *i*-th gene. The imputation is executed sequentially from the first gene of **G**_2_. That is, the first gene of **G**_2 _which has the smallest missing rate is selected as the target gene firstly. Then LLSimpute is applied to estimate the missing values in the target gene by finding the *k*-nearest neighbour genes from the complete matrix **G**_1 _and then using the formula in (9) to estimate the missing values. After filling all the missing values in the target gene, it is moved to **G**_1_. Then the second gene of **G**_2 _is selected as the target gene and repeat the same process again. By moving the genes whose missing values have been imputed to the complete matrix, the previous target genes with imputed values can be utilized for the missing value estimation of the following target gene. However, too many missing values in a gene will result in big estimation error and reusing a gene with too many imputed values will reduce the imputation performance. Therefore, only the genes with missing rates less than a threshold *r*_0 _are reused, where *r*_0 _is set as the average missing rate of all genes containing missing values, i.e.,

(13) $$r_{0}=\frac{\sum_{i=1}^{m-m_{1}}c_{i}}{\left(m-m_{1}\right)\times n}$$

By a similar argument as for the shrinkage LLSimpute, we apply the shrinkage estimator to SLLSimpute. The shrinkage SLLSimpute adjusts the coefficients of the regression model by the formula in (10) and use the formula in (11) to estimate the missing values.

### Shrinkage iterated local least squares imputation (Shrinkage ILLSimpute)

LLSimpute and SLLSimpute methods select *k*-nearest neighbor genes for a target gene, where *k *is a fixed number. However, in the ILLSimpute method [13], it does not fix the number of similar genes selected. Alternatively, it defines the similar genes as the genes whose distances to the target genes are less than a distance threshold δ . The rationale of using a distance threshold rather than using a fixed number of similar genes is that some of the *k*-nearest neighbor genes are already far away from the target gene and are not very similar to the target gene.

The procedure of ILLSimpute is as follows. In the first iteration, missing values of each target gene are filled with the row average. Then a distance threshold δ  is used to select the similar genes of each target gene. Finally, LLSimpute method is used to estimate the missing values of each target gene. In the later iteration, ILLSimpute method uses the imputed results from the previous iteration to reselect the similar genes of each target gene (using the same distance threshold) and applies LLSimpute method to re-estimate the missing values.

By a similar argument as for the shrinkage LLSimpute, we apply the shrinkage estimator to ILLSimpute. The shrinkage ILLSimpute adjusts the coefficients of the regression model by the formula in (10) and use the formula in (11) to estimate the missing values.

## Results and Discussion

We conducted several experiments to compare the performances of our shrinkage regression-based methods and the original regression-based methods under different scenarios. In the first subsection, we introduce the benchmark datasets. In the second subsection, we describe how we measure the performance of various imputation methods. In the following three subsections, we report the comparison results for different number of similar genes used, different missing rates, and different noise levels. Finally, we further compare the performances of our shrinkage regressioni-based methods and three existing non-regression-based methods.

### Datasets

Considering the effects of dataset selection and types of microarray experiments on the performance of an imputation method, six representative datasets (three non-time series and three time series) were used in our simulations. They were Ogawa's data from the study of phosphophate accumulation and poly-phosphophate metabolism (denoted as Ogawa, non-time series) [26], Bohen's follicular lymphomas data (denoted as BohenSH, non-time series) [27], the data from a lymphoma study (denoted as Lymphoma, non-time series) [28], the data from Brauer's experiments which studied the physiological response to glucose limitation in batch and steady-state cultures of yeasts (denoted as Brauer05, time series) [29], and Shapira's oxidative stress data (denoted as Shapira04A and Shapira04B, time series) [30]. We divided Shapira's data into two datasets because the authors used one kind of oxidative chemical in the experiment in Shapira04A, but they used another kind of oxidative chemical in the experiment in Shapira04B. The six microarray datasets were used as benchmark datasets in numerical experiments to compare the performances of our shrinkage regression-based methods and the original regression-based methods. Each dataset was processed by deleting the genes with missing values to generate a complete data matrix, and the details of these datasets were listed in Table 1.

**Table 1:** Benchmark datasets.

Name	Dimension of original datasets	Dimension of reduced complete datasets	Time series data	**Ref**.
Ogawa	6263 × 8	3069 × 8	N	[26]
BohenSH	2364 × 24	623 × 24	N	[27]
Lymphoma	4096 × 96	854 × 96	N	[28]
Brauer05	6133 × 20	706 × 20	Y	[29]
Shapira04A	4771 × 23	2970 × 23	Y	[30]
Shapira04B	4771 × 14	3340 × 14	Y	[30]

### The performance measure

A common criterion used to compare the performances of different imputation methods is the normalized root mean squared error (NRMSE) [11-13,17-19]. From a microarray dataset, we can obtain an original data matrix **M**_0 _with *m *genes and *n *experiments, and then we can construct a complete matrix $M_{1}\in {\mathbb{R}}^{m_{1}\;\times \;n}\left(m_{1}\leq m\right)$ by deleting the genes with missing values. After the complete data matrix **M**_1 _is established, we randomly select a specific percentage of the elements of **M**_1 _and regard these elements as missing values. Then we estimate the missing values using various imputation methods and compare their performances using NRMSE which is shown below:

(14) $$NRMSE=\frac{\sqrt{mean\left[{\left({\text{y}}_{\text{guess}}-{\text{y}}_{\text{ans}}\right)}^{2}\right]}}{std\left({\text{y}}_{\text{ans}}\right)}$$

where y_guess _and y_ans _are vectors whose elements are the estimated values by an imputation method and the known answers for all missing entries, respectively.

### Performance comparison for different *k *values

A parameter *k*, the number of similar genes used, has to be determined before using two regression-based methods (LLSimpute and SLLSimpute). Since the performance of both algorithms is known to be affected by the *k *value used and different microarray datasets may have different optimal *k *values [18,19], we tested several possible *k *values (50, 100, 150, 200, 250 and 300) on six benchmark datasets. Table 2 listed the optimal *k *values for LLSimpute and SLLSimpute on each of the six benchmark datasets. Another regression-based method (ILLSimpute) does not have the parameter *k *and therefore was not considered in this numerical experiment.

**Table 2:** The optimal *k *value for each benchmark dataset.

Algorithms\Datasets	Ogawa	BohenSH	Lymphoma	Brauer05	Shapira04A	Shapira04B
LLS	100	250	300	300	250	200
SLLS	150	300	250	300	250	200

For each of the six benchmark dataset, we also compared the performances of the proposed shrinkage regression-based methods and the original regression-based methods for several possible *k *values (50, 100, 150, 200, 250 and 300). In our numerical experiments, missing rate for each benchmark dataset was set to be 5%. Namely, for each dataset, we randomly removed 5% entries of the complete matrix to generate a matrix with missing values, and then estimated the missing values using the shrinkage and the original regression-based methods. The same procedure was run for five independent rounds and the average NRMSE of these five simulations was used to compare the performances of different imputation methods.

**Figure 2:**
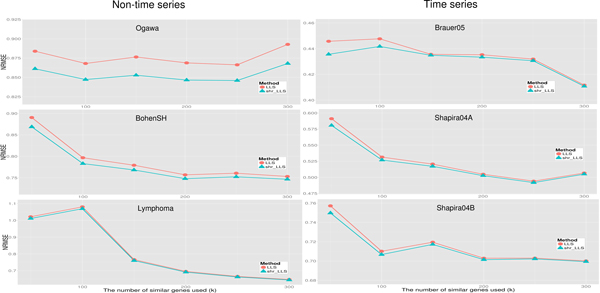
**Performance comparison between shrinkage LLS (shr_LLS) and LLS for different *k *values**.

**Figure 3:**
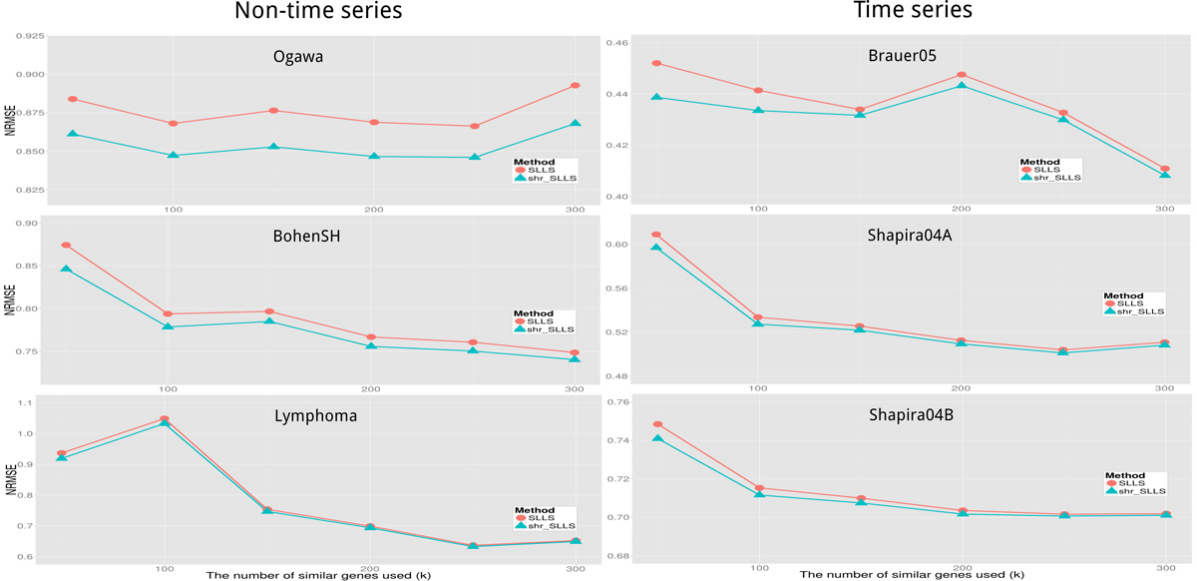
**Performance comparison between shrinkage SLLS (shr_SLLS) and SLLS for different *K *values**.

As shown in Figure 2, the proposed shrinkage LLSimpute outperforms LLSimpute for all *k *values and all benchmark datasets. Similarly, the proposed shrinkage SLLSimpute outperforms SLLSimpute for all *k *values and all benchmark datasets (see Figure 3). The simulation results suggest that utilizing a shrinkage estimation approach to adjust the coefficients of the regression model can improve the performances of the original regression-based methods.

### Performance comparison for different missing rates

In real applications, different microarray data may have different missing rates to be imputed. It is informative to know how an imputation method performs for different missing rates. Therefore, we compared the performances of the shrinkage regression-based methods and the original regression-based methods on the microarray data with different missing rates (1%, 5%, 10%, 15% and 20%). Namely, for each of the six benchmark dataset, we randomly removed *x*% (*x *= 1, 5, 10, 15 or 20) entries of the complete matrix to generate a matrix with missing values, and then estimated the missing values using the shrinkage and the original regression-based methods. The same procedure was run for five independent rounds and the average NRMSE of these five simulations was used to compare the performances of different imputation methods. Note that the optimal *k *value used for each benchmark dataset was listed in Table 2.

**Figure 4:**
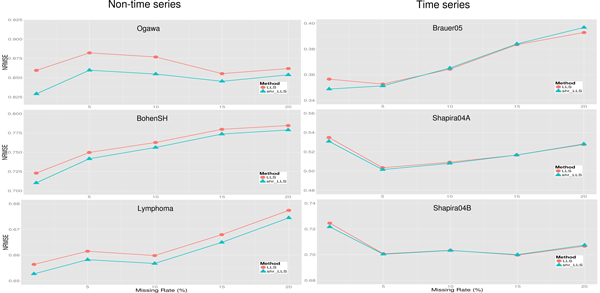
**Performance comparison between shrinkage LLS (shr_LLS) and LLS for different missing rates**.

**Figure 5:**
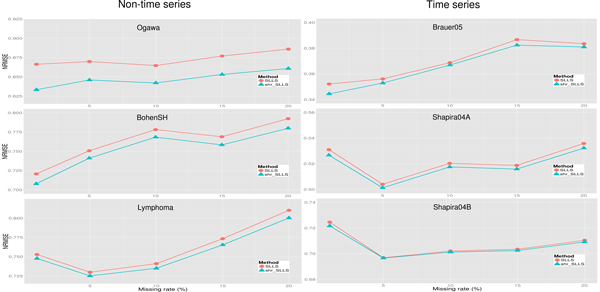
**Performance comparison between shrinkage SLLS (shr_SLLS) and SLLS for different missing rates**.

**Figure 6:**
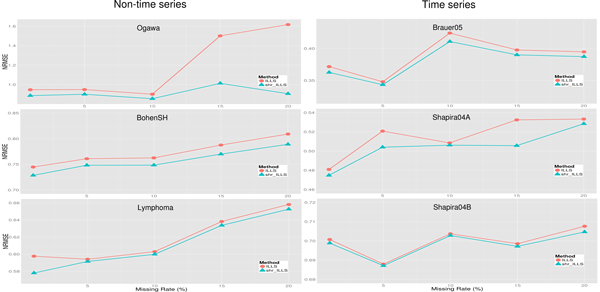
**Performance comparison between shrinkage ILLS (shr_ILLS) and ILLS for different missing rates**.

Figure 4 shows that the proposed shrinkage LLSimpute outperforms LLSimpute for all missing rates and all benchmark datasets. Figure 5 shows that the proposed shrinkage SLLSimpute outperforms SLLSimpute for all missing rates and all benchmark datasets. Figure 6 shows that the proposed shrinkage ILLSimpute outperforms ILLSimpute for all missing rates and all benchmark datasets. The simulation results suggest that utilizing a shrinkage estimation approach to adjust the coefficients of the regression model can improve the performances of the original regression-based methods.

### Performance comparison for different noise levels

In real applications, different microarray data may contain different levels of noises. It is informative to know how an imputation method performs for different levels of noises inherent in the microarray data. Therefore, we compared the performances of the shrinkage regression-based methods and the original regression-based methods on the microarray data with different noise levels. For each of the six benchmark dataset, we added Gaussian noises with different levels into the data. The magnitudes of the noises were set in terms of the standard deviations ranging from 0 to 0.25 with a step size 0.05. In our numerical experiments, missing rate for each benchmark dataset was set to be 5% and the optimal *k *value used for each benchmark dataset was listed in Table 2. Namely, for each dataset (after adding Gaussian noises into the data), we randomly removed 5% entries of the complete matrix to generate a matrix with missing values, and then estimated the missing values using the shrinkage and the original regression-based methods. The same procedure was run for five independent rounds and the average NRMSE of these five simulations was used to compare the performance of different imputation methods.

**Figure 7:**
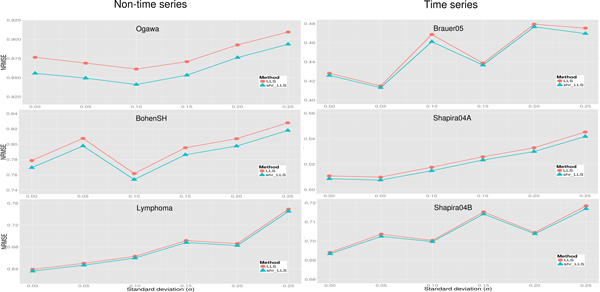
**Performance comparison between shrinkage LLS (shr_LLS) and LLS for different noise levels**.

**Figure 8:**
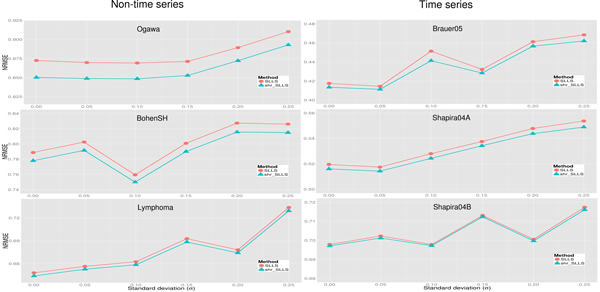
**Performance comparison between shrinkage SLLS (shr_SLLS) and SLLS for different noise levels**.

**Figure 9:**
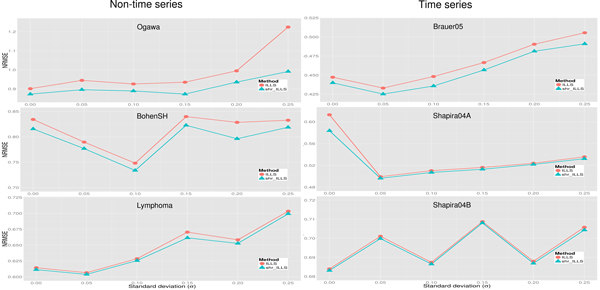
**Performance comparison between shrinkage ILLS (shr_ILLS) and ILLS for different noise levels**.

Figure 7 shows that the proposed shrinkage LLSimpute outperforms LLSimpute for all noise levels and all benchmark datasets. Figure 8 shows that the proposed shrinkage SLLSimpute outperforms SLLSimpute for all noise levels and all benchmark datasets. Figure 9 shows that the proposed shrinkage ILLSimpute outperforms ILLSimpute for all noise levels and all benchmark datasets. The simulation results suggest that utilizing a shrinkage estimation approach to adjust the coefficients of the regression model can improve the performances of the original regression-based methods.

### Performance comparison with three existing non-regression-based methods

**Figure 10:**
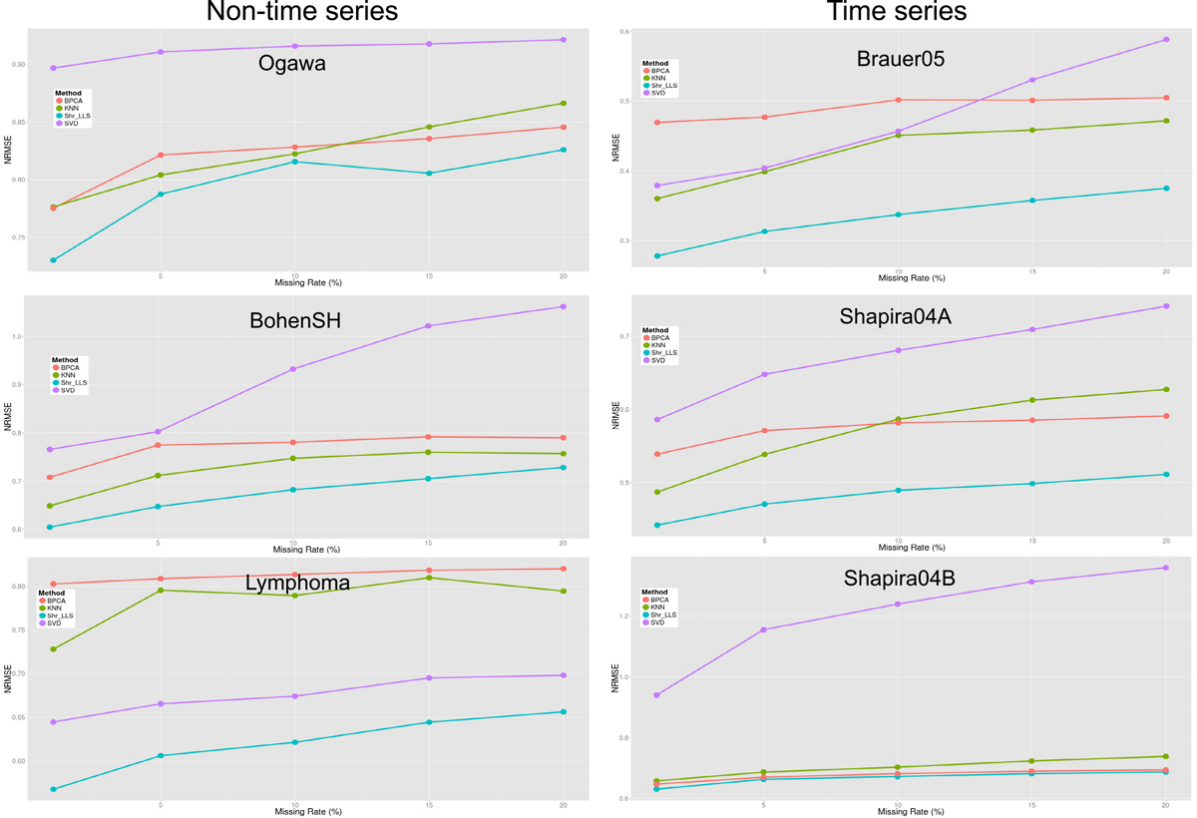
**Performance comparison between shrinkage LLS (shr_LLS) and three non-regression-based methods for different missing rates**.

**Figure 11:**
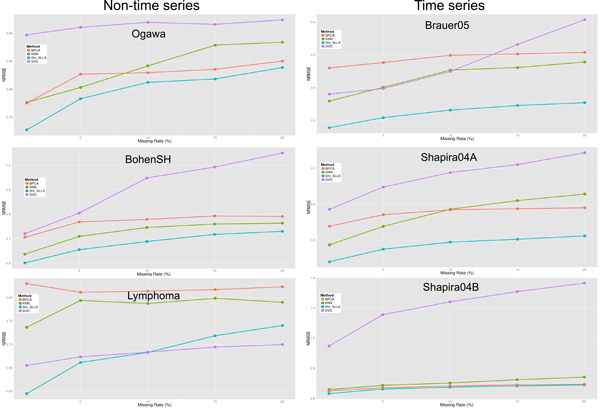
**Performance comparison between shrinkage SLLS (shr_SLLS) and three non-regression-based methods for different missing rates**.

**Figure 12:**
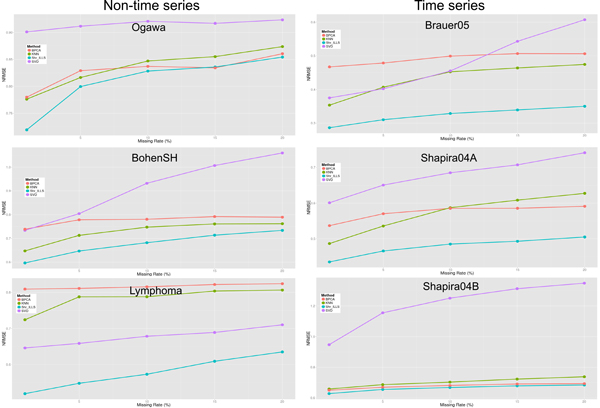
**Performance comparison between shrinkage ILLS (shr_ILLS) and three non-regression-based methods for different missing rates**.

We have shown that our shrinkage regression-based methods perform better than the existing regression-based methods. Still, it would be interesting to know whether our shrinkage regression-based methods provide more accurate missing value imputation than the existing non-regression-based methods do. Therefore, we compared the performances of our shrinkage regression-based methods and three existing non-regression-based methods (kNNimpute [12], SVDimpute [12], and BPCA [14]) on the six benchmark microarray datasets. As shown in Figures 10, 11, 12, the proposed shrinkage regression-based methods outperform these three existing non-regression-based methods for almost all missing rates and all benchmark datasets. Taken together, our shrinkage regression-based methods are competitive alternatives to the existing methods for microarray missing value imputation.

## Conclusions

Imputation of missing values is a very important aspect of microarray data analyses because most of downstream analyses require a complete dataset. Therefore, exploring accurate and efficient methods for estimating missing values has become an essential issue. In this study, regression-based methods associated with a shrinkage estimation approach are proposed to estimate missing values in the microarray data. Our methods take the advantage of the correlation structure existing in the microarray data and select similar genes for the target gene by Pearson correlation coefficients. Besides, our methods incorporate the least squares principle, utilize a shrinkage estimation approach to adjust the coefficients of the regression model, and apply the new coefficients of the regression model to estimate missing values. Simulation results show that the proposed shrinkage regression-based methods provide more accurate missing value estimation for various types of datasets than the original regression-based methods do. Since our proposed methods can be applied to modify any kind of regression-based methods and can provide accurate missing value estimation, they are competitive alternatives to the existing regression-based methods.

## Competing interests

The authors declare that they have no competing interests.

## Authors' contributions

WSW conceived the research topic and provided essential guidance. HW developed the alogirthm. CCC did all the simulations. HW, CCC, YCW, and WSW wrote the manuscript. All authors have read and approved the final manuscript.

## Declarations

The full funding for the publication fee came from Taiwan National Science Council and College of Electrical Engineering and Computer Science, National Cheng Kung University.

This article has been published as part of *BMC Systems Biology *Volume 7 Supplement 6, 2013: Selected articles from the 24th International Conference on Genome Informatics (GIW2013). The full contents of the supplement are available online at http://www.biomedcentral.com/bmcsystbiol/supplements/7/S6.
